# Myelin contrast across lamina at 7T, ex-vivo and in-vivo dataset

**DOI:** 10.1016/j.dib.2016.06.058

**Published:** 2016-07-05

**Authors:** Alessio Fracasso, Susanne J. van Veluw, Fredy Visser, Peter R. Luijten, Wim Spliet, Jaco J.M. Zwanenburg, Serge O. Dumoulin, Natalia Petridou

**Affiliations:** a Experimental Psychology, Helmholtz institute, Utrecht University, Utrecht, Netherlands; b Radiology, Imaging Division, University Medical Center, Utrecht, Netherlands; c Spinoza Centre for Neuroimaging, Amsterdam, Netherlands; d Neurology, Brain Center Rudolf Magnus, University Medical Center, Utrecht, Netherlands; e Philips Medical Systems, Best, Netherlands; f Pathology, University Medical Center Utrecht, Utrecht, Netherlands

**Keywords:** Myelin, Laminar profiles, Stria of Gennari, Lines of Baillarger

## Abstract

In this article we report the complete data obtained in-vivo for the paper: “Lines of Baillarger in vivo and ex-vivo: myelin contrast across lamina at 7T MRI and histology” (Fracasso et al., 2015) [1]. Single participant data (4 participants) from the occipital lobe acquisition are reported for axial, coronal and sagittal slices; early visual area functional localization and laminar profiles are reported. Data from whole brain images are reported and described (5 participants), for axial, coronal and sagittal slices. Laminar profiles from occipital, parietal and frontal lobes are reported. The data reported in this manuscript complements the paper (Fracasso et al., 2015) [1] by providing the full set of results from the complete pool of participants, on a single-participant basis. Moreover, we provide histological images from the ex-vivo sample reported in Fracasso et al. (2015) [1].

**Specifications Table**

**Table:** 

Subject area	*Neuroscience*
More specific subject area	*High-resolution MRI, myelination across cortical thickness*
Type of data	*Figures, plots, table*
How data was acquired	*Structural and functional MRI, 7T*
Data format	*Raw*
Experimental factors	*Retinotopy data are shown as color overlays depicting early visual cortex areas V1, V2, V3 over cortex surface; Raw images were motion corrected, averaged, underwent proton density correction to correct for inhomogeneities;*
Experimental features	Participants were asked to lie still in the scanner while multiple anatomical acquisitions (T1-weighted images) were acquired at high resolution (0.5 isotropic). For the retinotopy data, participants were asked to lie still in the scanner while keeping fixation in the center of the screen and being presented with a functional mapping stimuli. Multiple functional acquisitions (T2*-weighted images) were acquired.
Data source location	*Utrecht, The Netherlands*
Data accessibility	*Data is with this article.*

**Value of the data**

• This data shows the presence of a highly myelinated band throughout the human cortex in multiple participants, across scans.
• The data could be used to compare results obtained across different scanners at ultra-high field.
• The data could be used to compare results obtained across different sequences at ultra-high field.

## 1 Data

The data consists of T1-weighted images from occipital lobe and whole-brain scans from multiple views (axial, sagittal and coronal), as well as T1-w intensity laminar profiles from the white matter border to cerebro-spinal fluid border for a number of locations in the occipital, parietal and frontal lobes.

T1-w data from the occipital lobe is coupled with functional retinotopic mapping (via population receptive field estimation) to evaluate the overlap of T1-w intensity laminar profiles with functionally defined early visual cortex areas. We also provide a set of histological images from the ex-vivo sample reported in [1].

## 2 Experimental design, materials and methods

Four males participated in the in vivo experiment occipital lobe acquisition, and five males participated in the in vivo experiment whole brain acquisition (age range 25–39 years). All experimental procedures were conducted in accordance with the 1964 Declaration of Helsinki (most recently amended in 2008, Seoul), and approved by the ethics committee of the UMCU. Images were obtained using a similar T1-w MPRAGE sequence as used for the ex-vivo scan, optimized for in vivo imaging. A strong contrast between gray and white matter was obtained with time delay (TD)=6 s (approximately 3 times the T1 of GM, allowing full recovery of both GM and white matter (WM) prior to inversion) and inversion delay (TI)=1200 ms (yielding GM signal just above zero). At this TI and TD, cerebrospinal fluid (CSF) appears bright in the reconstructed magnitude images. Occipital and whole-brain images were acquired per subject, as described below. A proton density (PD) scan centered on the occipital and whole brain field-of-view (FOV), respectively, was acquired for subsequent correction of B1 field inhomogeneities and for correction of the intensity inhomogeneity due to the sensitivity profiles of the receive coils.

### 2.1 Occipital images

The FOV was centered on the occipital pole. Acquisition parameters were: TD/TI: 6000/1200 ms, TR/TE: 8/4 ms, flip angle: 8°, adiabatic inversion, voxel size: 500×500×500 μm^3^, FOV: 140×140×30 mm^3^, 60 coronal slices, bandwidth 202 Hz/px, number of excitations per inversion: 275, linear readout, and no acceleration. Scan duration was ~7.5 min, 6 repeated scans were acquired per participant. This protocol was adopted to increase the signal-to-noise ratio (which would be compromised with the use of accelerated imaging) at the cost of scan time duration. PD scan parameters: 3D turbo-field echo, TR/TE: 9/2 ms, FOV: 140×140×30 mm, flip angle=1°, voxel size: 1×1×1 mm^3^, 30 coronal slices, BW: 506 Hz/px, no acceleration, scan duration=26 s.

### 2.2 Whole-brain images

The whole brain protocol included acceleration to investigate the feasibility of the technique within clinically acceptable scan times. Whole brain coverage was obtained with the following parameters: TD/TI: 6000/1200 ms, TR/TE: 8/3 ms, (repetition time and echo time, respectively), flip angle: 8°, adiabatic inversion, voxel size=500×500×500 μm^3^, FOV (field of view): 250×250×180 mm^3^, 360 sagittal slices, BW (bandwidth) 201 Hz/px, number of excitations per inversion: 300, linear readout, acceleration using SENSE: 2.5 (anterior–posterior)×2.5(right–left). Additionally, we used an optimized linear sampling scheme over ky and kz provided by the vendor software (termed Free factor), effectively allowing a high number of excitations per inversion in combination with an elliptical k-space shutter. The elliptical k-space shutter does not acquire the corner regions of k-space [2]. Scan duration was 7.5 min (4 to 5 repetitions were acquired for each participant, scan duration without the use of Free factor would be ~15 min). PD scan parameters: 3D turbo-field echo, TR/TE: 6/3 ms, FOV: 250×250×180 mm, voxel size: 1×1×1 mm^3^, flip angle 1°, 180 sagittal slices, BW: 253 Hz/px, SENSE: 1.8(anterior–posterior)×1.8(right–left), scan duration 48 s. The same sequence was used to re-acquire the data from one participant with a different resolution (voxel size=600×600×600 μm^3^) with all other parameters being the same, as a control for the presence of truncation artifacts.

### 2.3 Preprocessing

Here we provide a description of how motion correction was performed and how we obtained homogeneous image intensity in the acquired images.

in vivo occipital and whole-brain T1-w images were corrected for head movement between scan acquisitions using the 3dAllineate function in AFNI (http://afni.nimh.nih.gov), using 6 degrees of freedom and imposing wsinc5 as the last interpolation step (Table 1). After motion correction, images of the same FOV were averaged together to increase SNR. PD images were resampled to the corresponding T1-w space and blurred with a Gaussian smoothing kernel (full width half-max=12 mm). T1-w images were divided by the blurred PD images of the corresponding FOV to correct for field inhomogeneities [3]. Subsequently, data were resampled for laminar analysis and for surface analysis (the latter only for whole brain images).

### 2.4 Location selection

Four locations were selected from the occipital T1-w images for each participant: two in the calcarine cortex and two extra-calcarine locations.

On the whole brain T1-w images, we selected 4 similar locations for each hemisphere per participant, in the occipital, parietal and frontal lobes (total of 12 separate locations for each participant). Regions were arbitrarily selected throughout the brain, selecting similar locations for the two hemispheres.

### 2.5 Laminar analysis

Here we describe our operational definition of an intensity profile and how we obtained the single intensity profiles across cortical depth from the selected locations.

For each location the volume was manually segmented using Slicer [4], delineating WM and the CSF . The segmented WM and CSF were smoothed with a Gaussian kernel (std=0.8 voxels). The faces and vertices of the WM and CSF meshes were extracted from the isosurface of the volume (using the MATLAB function isosurface). The normals of the white mesh were computed and grown until they intersected the CSF mesh. Only the normals that intersected the CSF mesh were kept for subsequent analysis (valid normals), all the other normals were not analyzed further. Operational definition of intensity profile: a single profile was obtained by interpolating (via nearest neighbor interpolation) the volume along the valid normals until reaching the CSF mesh in 100 steps.

The Euclidean distance with respect to GM–WM border was computed (equi-distance approach). Waehnert et al. [5] showed that an equi-volume approach in estimating layer 4 location outperformed the equi-distance approach for extremely high acquisition resolutions (~0.1 mm isotropic). However, they also showed that the equi-volume and equi-distance approaches were indistinguishable at relatively lower resolutions (0.5 mm isotropic).

**Fig. 1:**
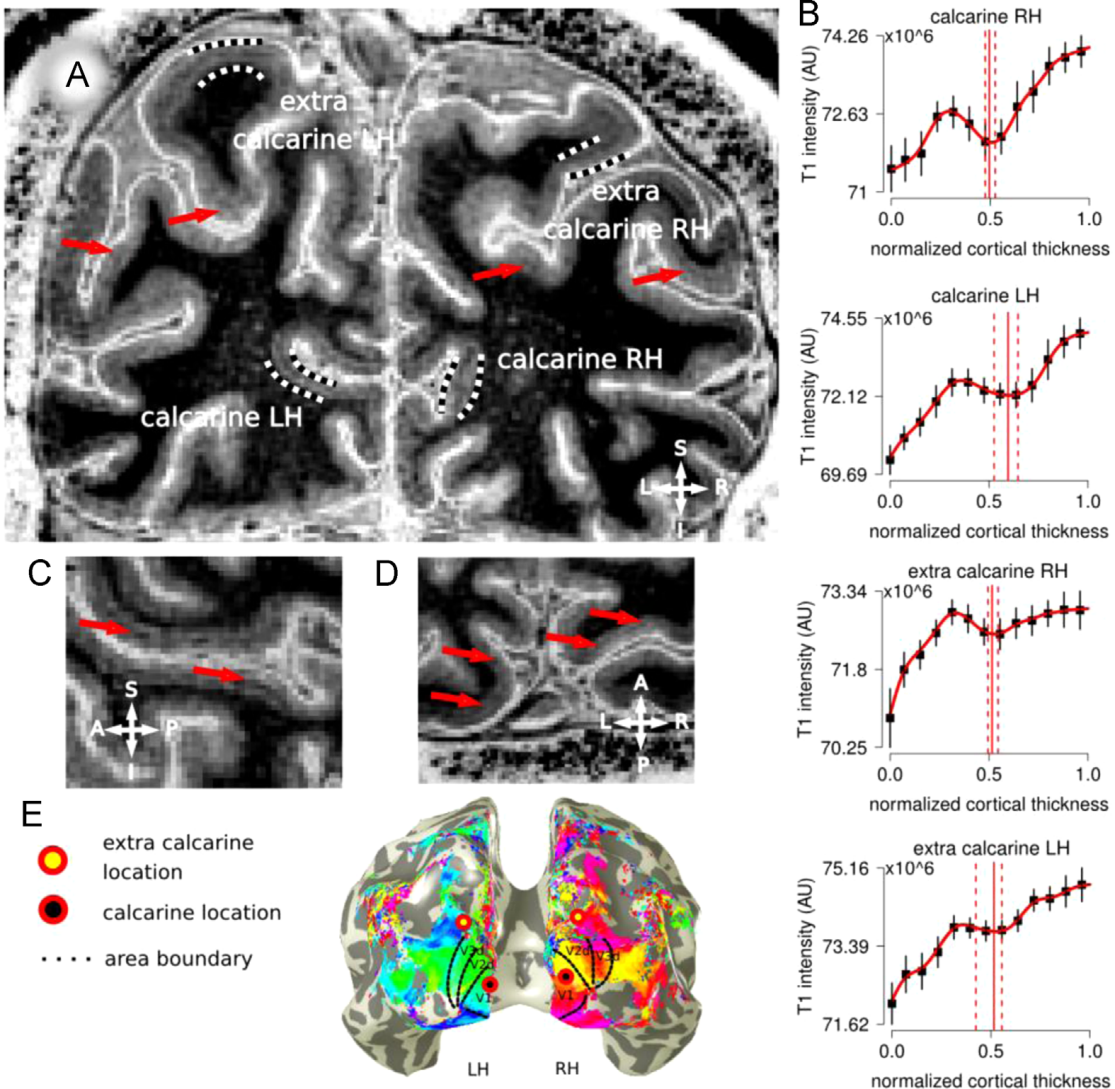
Panel A, in vivo T1-w image for one participant (P1). Locations in the calcarine cortex and extra-calcarine cortex are indicated in the image (black white dotted lines). Red arrows point to other locations where an hypo-intense band is visible around the middle of cortical thickness. Panel B, Laminar profiles are reported from the GM/WM border (0 on the *x*-axis) to the GM/CSF surface (1 on the *x*-axis) for each location. Error bars indicate the standard deviation over all profiles for each location, red lines in graphs represent median and bootstrapped 95% CI of the hypo-intense band location. A T1-w hypo-intense band was identified in more that 75% of the bootstrapped profiles (see ׳Laminar data analysis׳) in calcarine and extra-calcarine locations. Panel C, sagittal view; Panel D, axial view, centered on the calcarine; Panel E, early visual area retinotopic maps for participant 1, superimposed to the reconstructed whole brain 3D mesh from the conventional T1-w anatomical MRI. Areas V1 to V3 are reported. Black dots with a red surround represent calcarine locations projected to the mesh surface, yellow dots with a red surround represent extra-calcarine locations projected to the mesh surface. Extra-calcarine locations are well outside the functionally defined area V1. 3 The datasets for the other two participants follow (results from participant 1 are reported in [1]).

For each location a variable number of individual profiles was obtained, ranging from 25 to 45 profiles (please see description above for the operational definition of an intensity profile). All the profiles were averaged together (Fig. 1C, only 13 points are reported, the complete profile consisted of 100 points). Then, the average profile was smoothed using the R software (http://www.R-project.org) implementation of cubic spline fit with 0.55 as a smoothing parameter. The smoothing step was incorporated to low-pass the signal, in order to find a stable zero-crossing point in the first derivative of the profile, see below.

An estimate of a change in profile intensity along the average profile was derived by means of a bootstrapping procedure (2000 repetitions with replacement). A change in the profile intensity was defined as the first negative to positive zero-crossing point of the first derivative of the smoothed profile, extracted for each bootstrapped iteration, if it existed (hypo-intense band). For each iteration we extracted also the first positive to negative zero-crossing point of the first derivative, if it existed, giving an index of where the hypo-intense band started along the profile.

For any given location, we assessed whether the proportion of bootstrapped samples where an hypo-intense band was identified statistically exceeded 75%, using the test of given proportions. Multiple tests were corrected using Bonferroni correction. In case we could reject the null hypothesis that the proportion was smaller than or equal to 75%, a reliable hypo-intense band in profile intensity was identified for that specific location. Given that the *p*-value for the test of given proportions was computed based on the bootstrapped samples, we opted to report only the confidence interval of hypo-intense band, when present, rather than the *p*-value.

For any given location we assessed the amplitude of the hypo-intense band by subtracting the profile intensity at start of the hypo-intense band from the profile intensity at the minimum of the hypo-intense band.

### 2.6 Retinotopy: visual stimuli setup

Visual stimuli were presented by back-projection onto a 15.0×7.9 cm screen inside the MRI bore. Participants viewed the display through prisms and mirrors, and the total distance from the participant׳s eyes (in the scanner) to the display screen was 35 cm. Display resolution was 1024×538 pixels. The stimuli were generated in Matlab (Mathworks, Natick, MA, USA) using the PsychToolbox [6], [7].

### 2.7 Definition of early visual cortex areas, pRF modeling, and coregistration of occipital images

Early visual cortex maps (V1–V3, V3A and V3B) were reconstructed using near-identical procedures as in previous studies [8], [9], [10], [11]. Stimuli consisted of drifting bar apertures at four orientations, which exposed a checkerboard pattern moving parallel to the bar orientation [8]. Alternating rows of checks moved in opposite directions, and orthogonally with respect to the bar orientation. The bar width (and width of alternating white and black checks) subtended one-quarter of the stimulus radius (1.56° of visual angle). The bar moved across the stimulus aperture in 20 evenly spaced steps, each 0.625° of visual angle, 1/20th of the stimulus window diameter. As there was one step at the start of each functional volume acquisition, each pass of the stimulus lasted 20 acquisition repetitions (TRs), 30 s. Four bar orientations and two different motion directions for each bar were presented, giving a total of eight bar motion directions (upward, downward, left, right, and four diagonals) within each run (the same stimuli order was presented for each run). After each horizontal or vertical bar orientation pass, a 30 s of mean-luminance (zero contrast) stimulus was displayed. Four mean-luminance blocks were presented at regular intervals during the scan. Participants fixated on a dot in the center of the visual stimulus. We used a population receptive field (pRF) model to reconstruct visual field maps; the model estimates a pRF for every voxel using a method previously described [8]. We used the position estimates of the pRF to define the visual areas. Areas were defined on a conventional T1-w anatomical MRI data with a voxel size of 0.8×0.8×0.8 mm (resampled at 1 mm isotropic), TR of 7 ms, echo time (TE) of 2.84 ms, and flip angle of 8°. Anatomical data were acquired after the functional data acquisition. Functional T2*-weighted multi-slice echo-planar images (EPI) were acquired using a Philips Achieva 7T scanner (Best, Netherlands), a volume transmit coil for excitation and a 32-channel head coil for signal reception (Nova Medical, MA, USA). Acquisition parameters were: TR/TE: 1500/30 ms, flip angle: 70°, voxel size: 2 mm isotropic, and 24 coronal slices, centered on the occipital pole. Functional scans were each 248 time frames (372 s) in duration, and the first eight time frames were discarded to ensure that signal had reached steady state.

T1-w occipital images were coregistered with the T1-weighted anatomical scan acquired during the retinotopic mapping scans. The 3D anatomical mesh of the conventional T1-w was created in FreeSurfer and imported in mrVista (https://github.com/vistalab/vistasoft). The locations selected for the laminar analysis were projected over the reconstructed 3D anatomical mesh of the conventional T1-w anatomical MRI of each participant, to localize the position with respect to early visual cortex area borders.

#### 2.7.1 Dataset

Occipital lobe images and functional data, participant 2, (results from participant 1 are reported in [1]) .

**Fig. 2:**
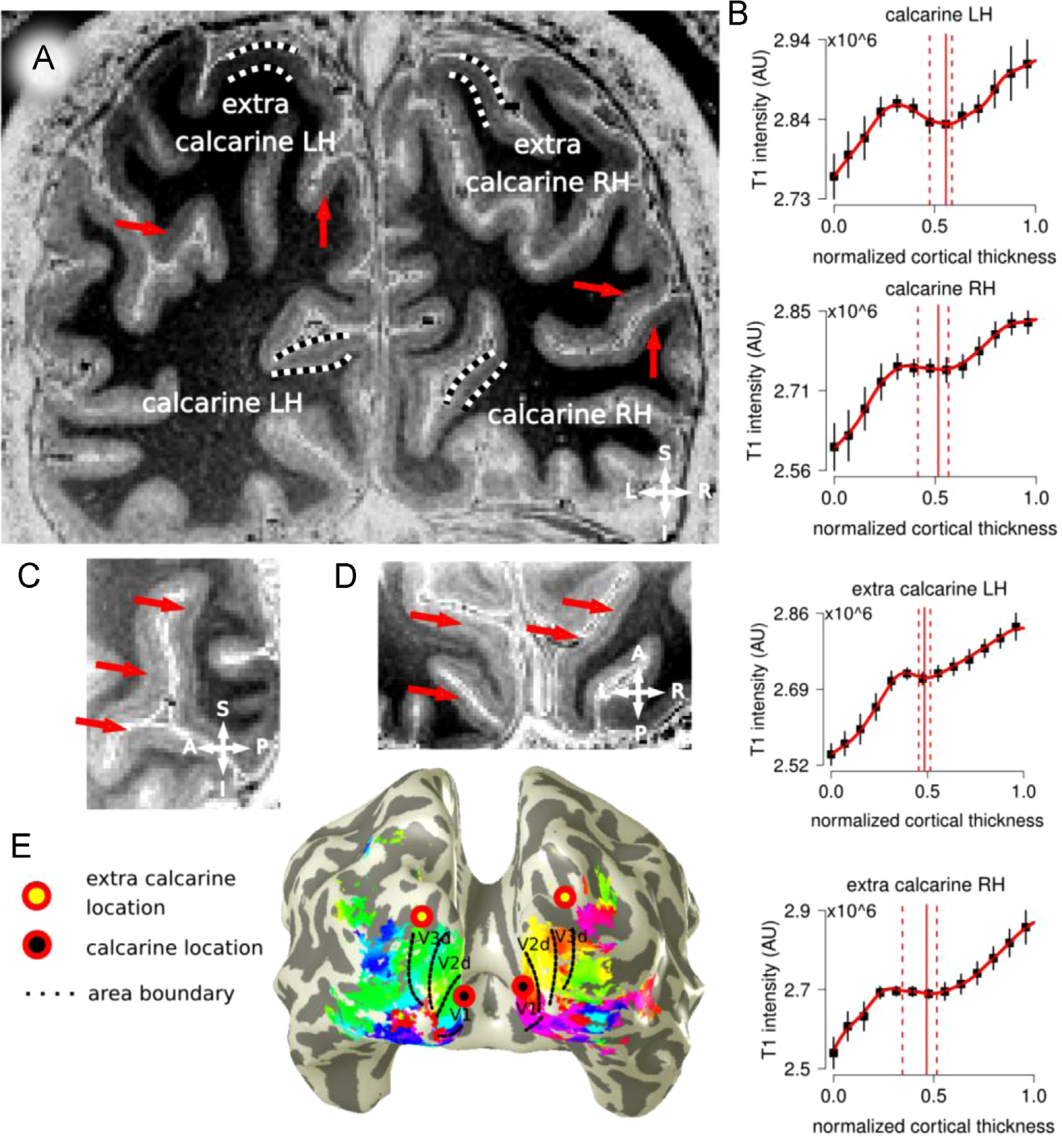
Panel A, in vivo T1-w image for participant 3 (P3). Locations in the calcarine cortex and extra-calcarine cortex are indicated. Panel B, Laminar profiles are reported from the GM/WM border (0 on the *x*-axis) to the GM/CSF surface (1 on the *x*-axis) for each location, error bars indicate the standard deviation over all profiles for each location. Panel C, sagittal view; Panel D, axial view, centered on the calcarine; Panel E, early visual area retinotopic maps for participant 3, superimposed to a reconstructed whole brain 3D mesh. Areas V1 to V3 are reported. For a full description please see the caption of Fig. 1.

Occipital lobe images and functional data, participant 3 (Fig. 2).

**Fig. 3:**
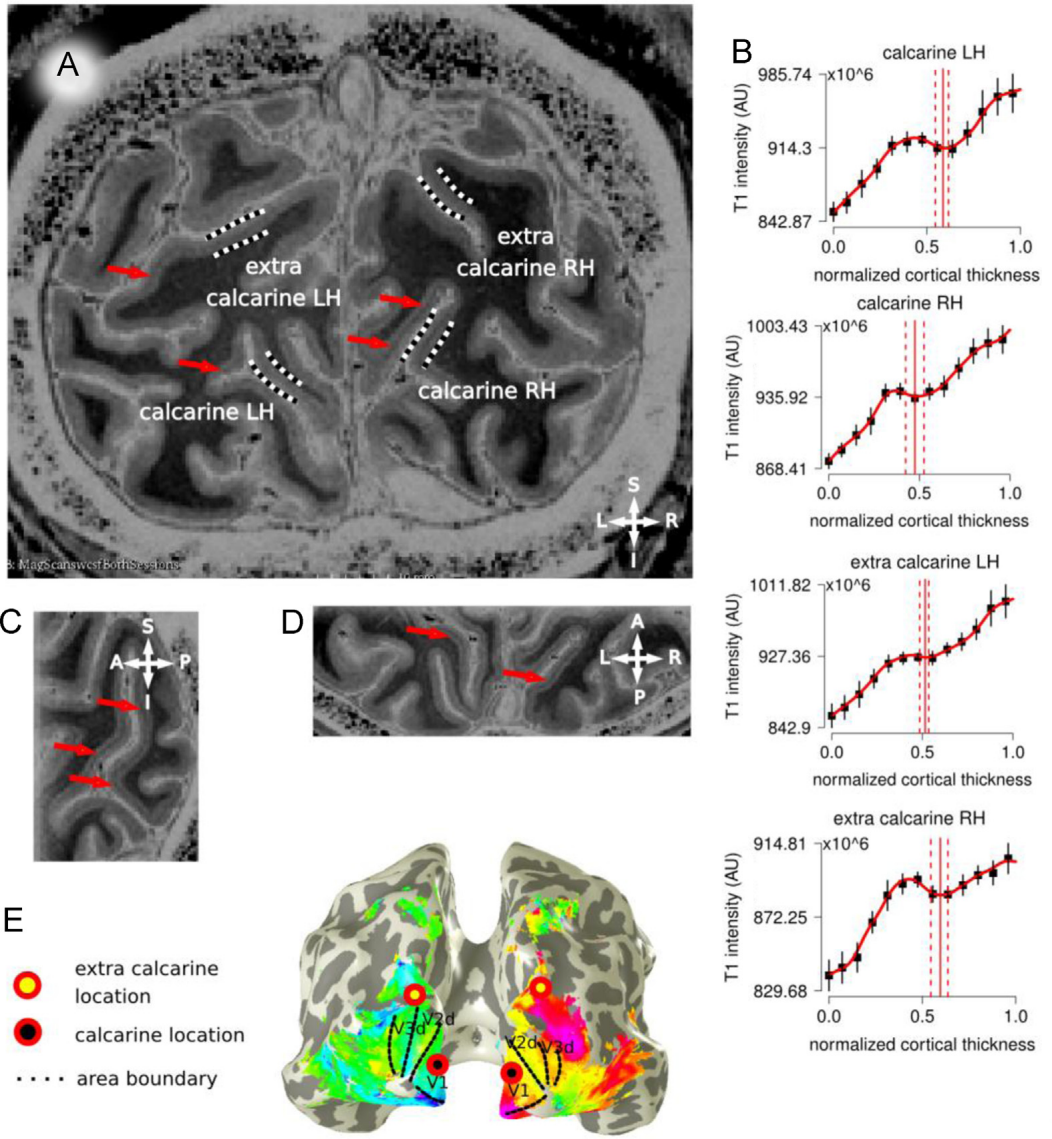
Panel A, in vivo T1-w image for participant 4 (P4). Locations in the calcarine cortex and extra-calcarine cortex are indicated. Panel B, Laminar profiles are reported from the GM/WM border (0 on the *x*-axis) to the GM/CSF surface (1 on the *x*-axis) for each location, error bars indicate the standard deviation over all profiles for each location. Panel C, sagittal view; Panel D, axial view, centered on the calcarine; Panel E, early visual area retinotopic maps for participant 4, superimposed to a reconstructed whole brain 3D mesh. Areas V1 to V3 are reported. For a full description please see the caption of Fig. 1.

Occipital lobe images and functional data, participant 4 (Fig. 3).

**Fig. 4:**
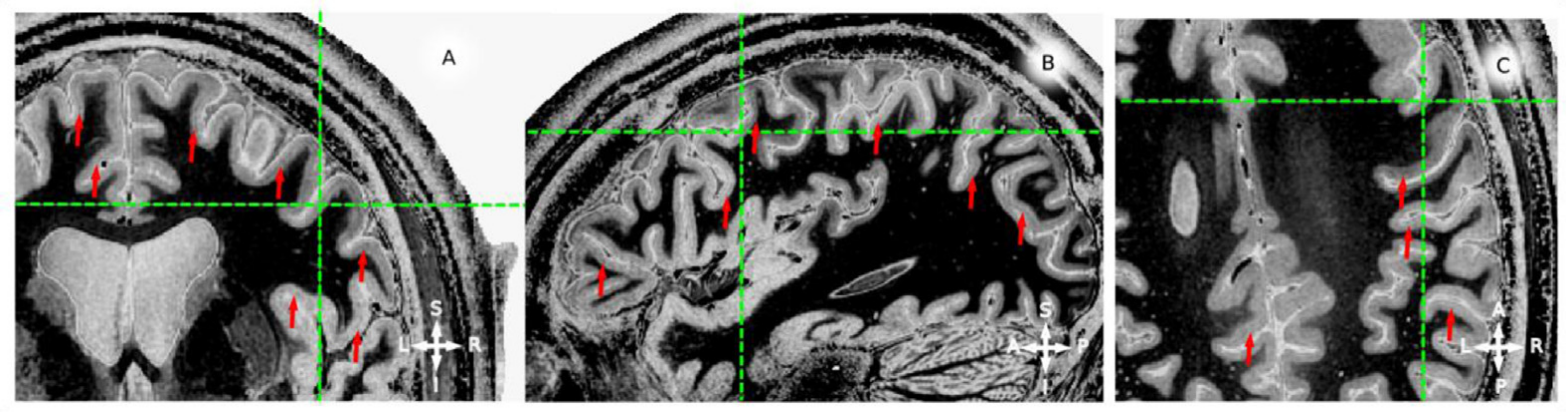
in vivo whole brain T1-w data, example coronal (panel A), sagittal (panel B) and axial (panel C) slices for participant 2 (P2). Red arrows point to locations where an hypo-intense band around the middle of cortical thickness is visible. Green lines mark the corresponding coordinate on the three views. Note that the hypo-intense *band* can be identified for both hemispheres and from posterior to anterior locations.

**Fig. 5:**
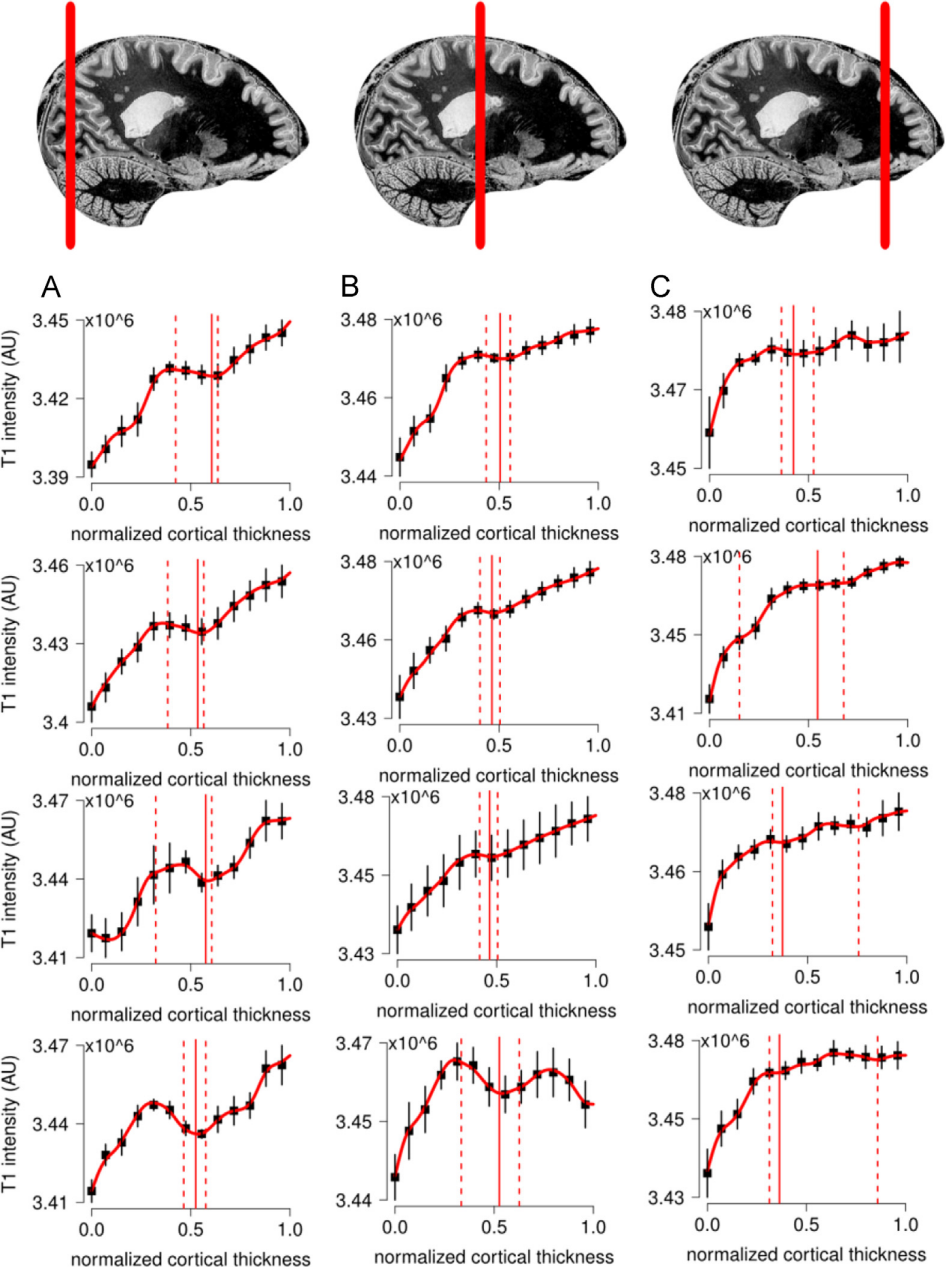
in vivo whole brain T1-w data for participant P2. Columns correspond to the occipital (A), parietal (B) and frontal (C) lobes as marked by the vertical red line in the sagittal view. Profiles are taken from similar locations in the left and right hemispheres. Laminar profiles are reported from the GM/WM border (0 on the *x*-axis) to the GM/CSF surface (1 on the *x*-axis) for each selected location. Error bars indicate standard deviation over all profiles for each location. A T1-w hypo-intense band was identified for all locations (vertical red lines in graphs represent median and bootstrapped 95% CI of hypo-intense band location).

Whole brain images, multiple views and profiles, participant 2 (results from participant 1 are reported in [1]) (Fig. 4, Fig. 5).

**Fig. 6:**
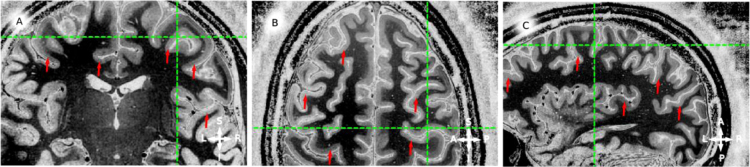
For the complete description see the caption of Fig. 4.

**Fig. 7:**
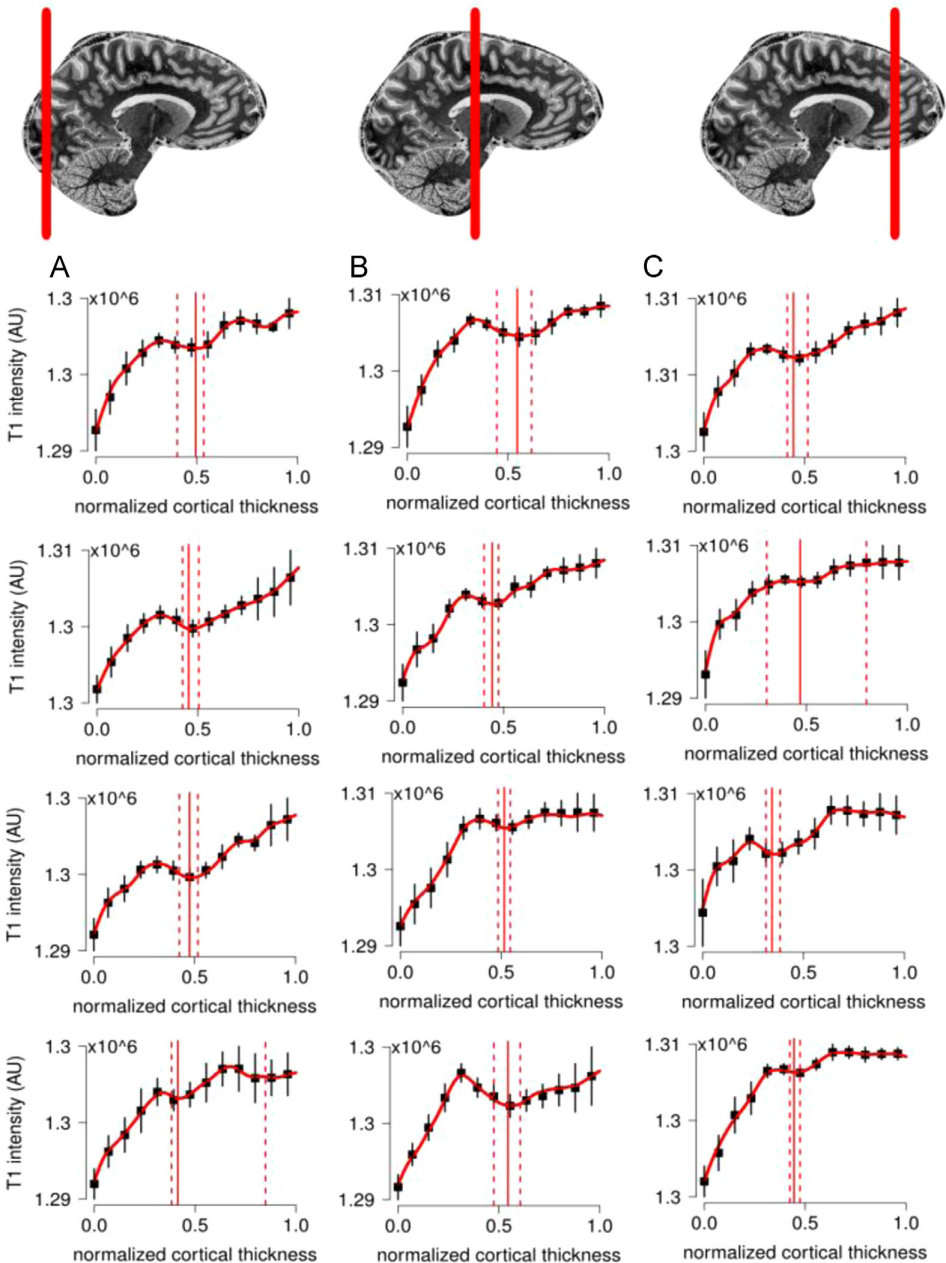
For the complete description see the caption of Fig. 5.

Whole brain images, multiple views and profiles, participant 3 (Fig. 6, Fig. 7).

**Fig. 8:**
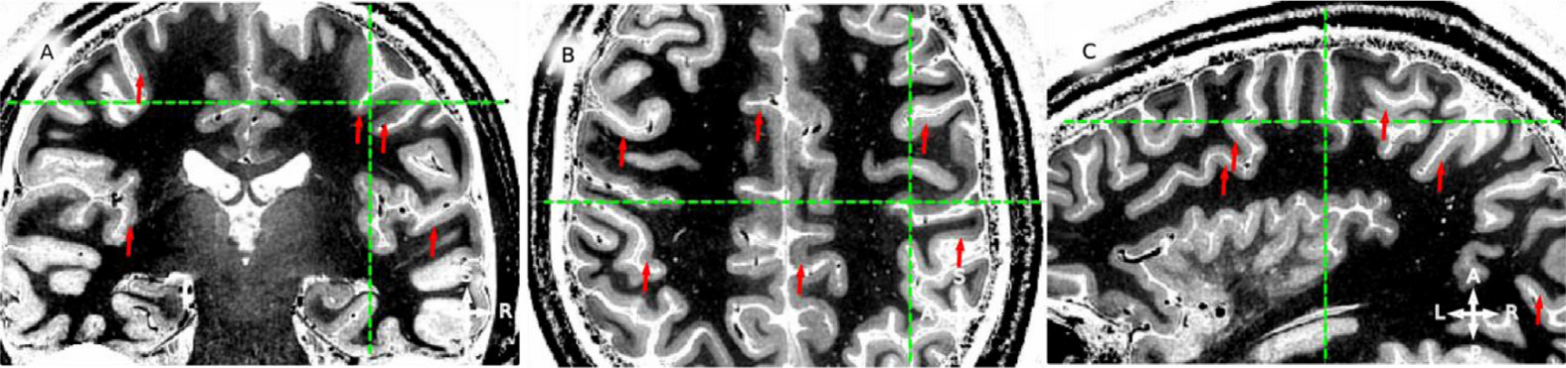
For the complete description see the caption of Fig. 4.

**Fig. 9:**
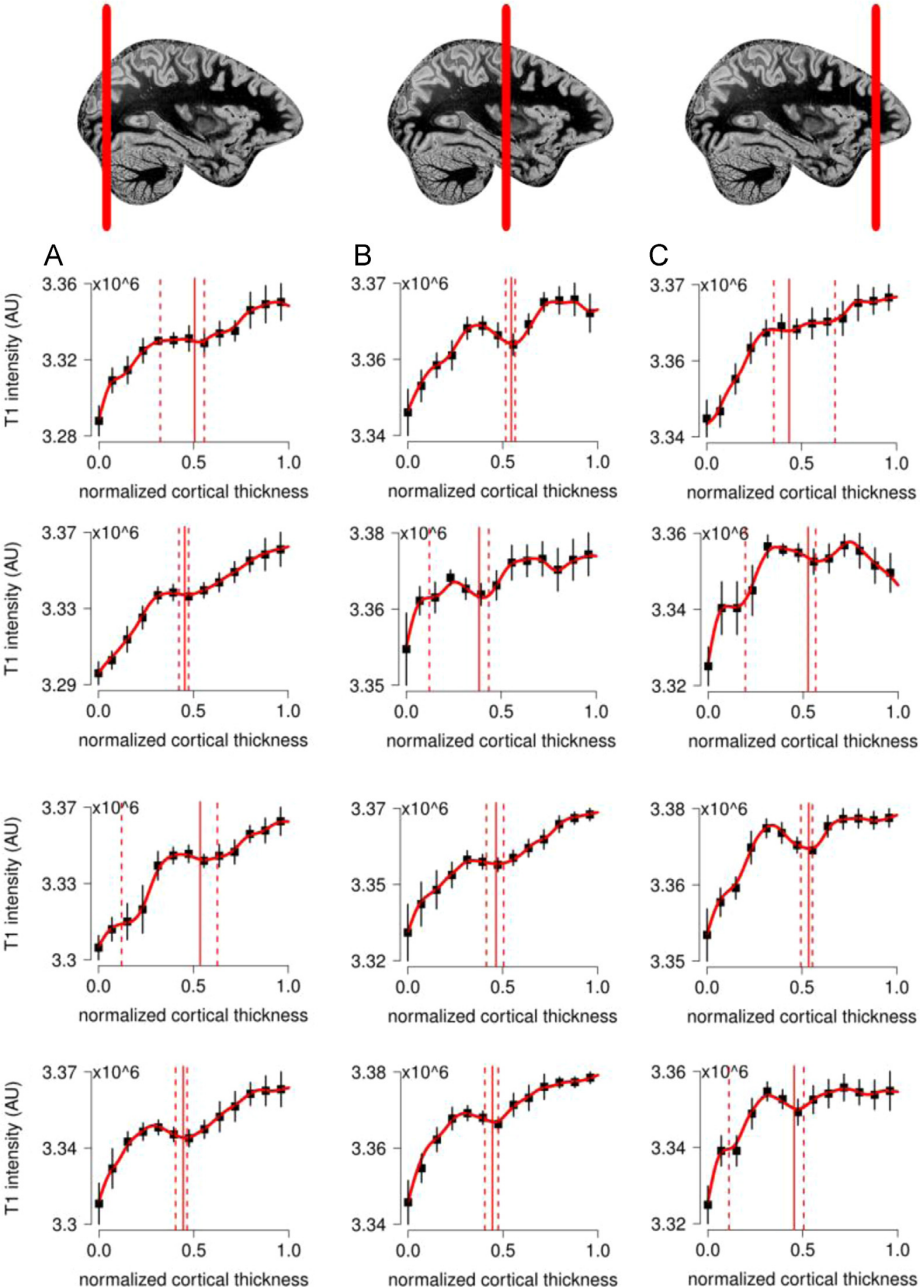
For the complete description see the caption of Fig. 5.

Whole brain images, multiple views and profiles, participant 4 (Fig. 8, Fig. 9).

**Fig. 10:**
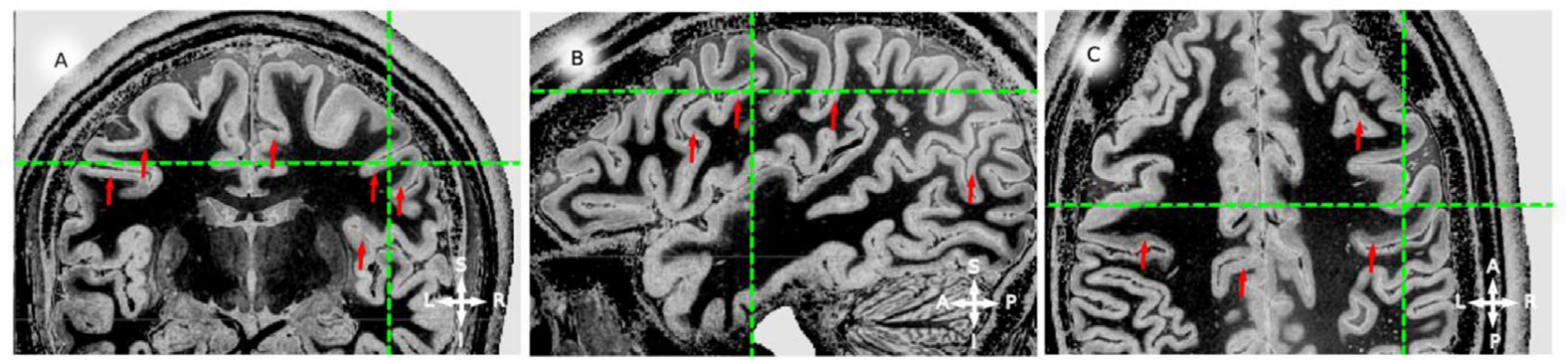
For the complete description see the caption of Fig. 4.

**Fig. 11:**
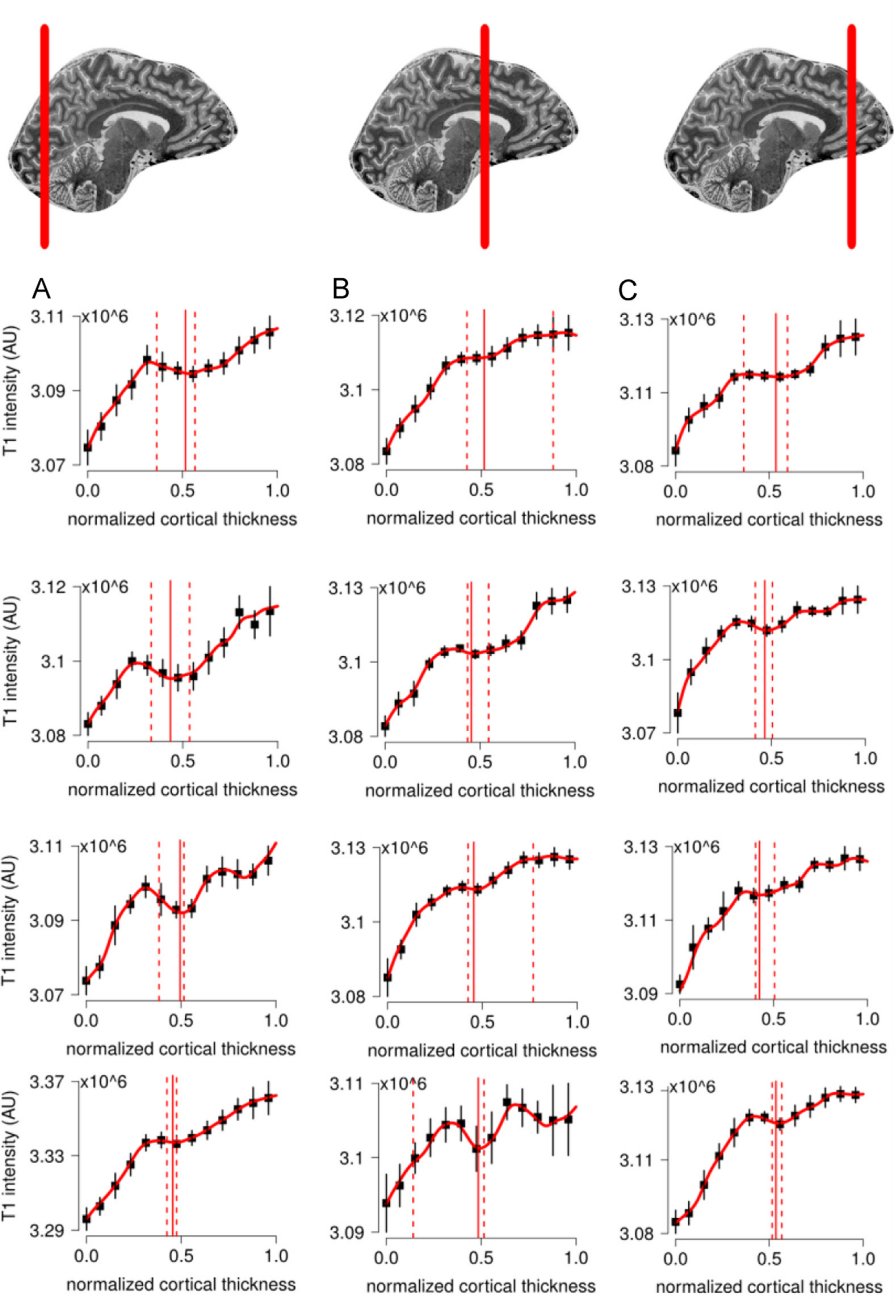
For the complete description see the caption of Fig. 5.

**Fig. 12:**
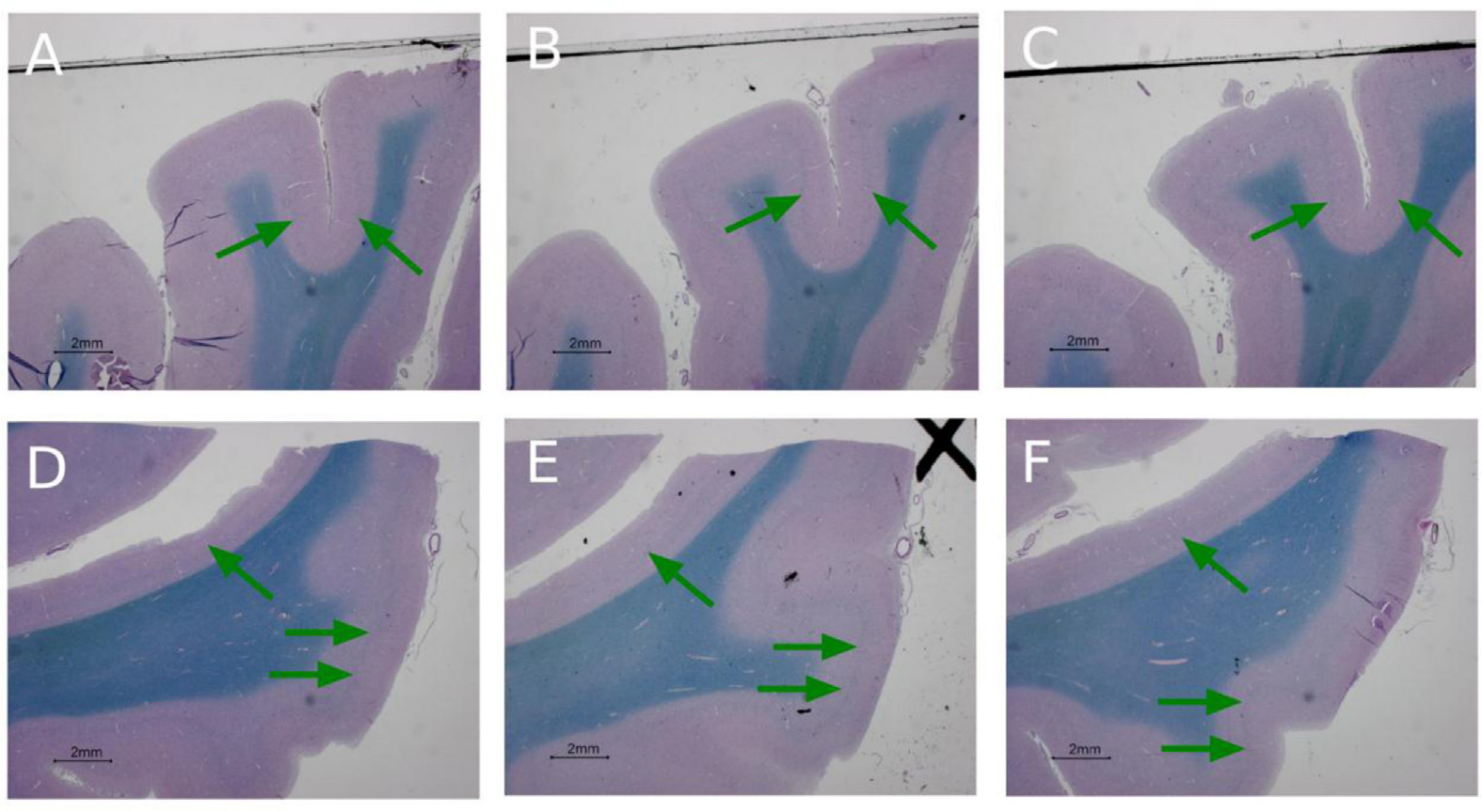
Example histological images (adjacent sections were stained with Luxol fast blue – Periodic Acid Schiff (LP) for the visualization/identification of myelin) panels A–C extra-calcarine histological samples, same location reported in Fig. 4D but different histological stains (Fracasso et al., 2016). Panels D–F, extra-calcarine histological samples, same location reported in Fig. 3D but different histological stains (Fracasso et al., 2016), green arrows point to locations where a highly-myelinated band is present.

Whole brain images, multiple views and profiles, participant 5 (Fig. 10, Fig. 11, Fig. 12).

## Figures and Tables

**Table 1:** Motion correction results for the occipital scan and whole brain scan. For each scan and participant the largest motion detected between multiple acquisitions is reported (in mm).

Occipital scan					
Participant	p1	p2	p3	p4	
Max motion (mm)	1.7	2.8	2.2	0.55	

Whole-brain scan					
Participant	p1	p2	p3	p4	p5
Max motion (mm)	0.45	0.57	0.83	0.91	1.5

## References

[1] Fracasso A., van Veluw S.J., Visser F., Luijten P., Spliet W., Zwanenburg J.J., Dumoulin S.O., Petridou N. (2015). Lines of Baillarger in vivo and ex vivo: myelin contrast across lamina at 7T MRI and histology. Neuroimage.

[2] Fair M.J., Gatehouse P.D., DiBella E.V., Firmin D.N. (2015). A review of 3D first-pass, whole-heart, myocardial perfusion cardiovascular magnetic resonance. J. Cardiovasc. Magn. Reson..

[3] Van de Moortele P.F., Auerbach E.J., Olman C., Yacoub E., Uğurbil K., Moeller S. (2009). T 1 weighted brain images at 7 Tesla unbiased for proton density, T 2* contrast and RF coil receive B 1 sensitivity with simultaneous vessel visualization. Neuroimage.

[4] Fedorov A., Beichel R., Kalpathy-Cramer J., Finet J., Fillion-Robin J.-C., Pujol S., Bauer C., Jennings D., Fennessy F., Sonka M., Buatti J., Aylward S., Miller J.V., Pieper S., Kikinis R. (2012). 3D Slicer as an image computing platform for the quantitative imaging network. Magn. Reson. Imaging.

[5] Waehnert M.D., Dinse J., Weiss M., Streicher M.N., Waehnert P., Geyer S., Bazin P.L. (2014). Anatomically motivated modeling of cortical laminae. Neuroimage.

[6] Brainard D.H. (1997). The psychophysics toolbox. Spat. Vis..

[7] Pelli D.G. (1997). The VideoToolbox software for visual psychophysics: transforming numbers into movies. Spat. Vis..

[8] Dumoulin S.O., Wandell B.A. (2008). Population receptive field estimates in human visual cortex. Neuroimage.

[9] Amano K., Wandell B.A., Dumoulin S.O. (2009). Visual field maps, population receptive field sizes, and visual field coverage in the human MT+ complex. J. Neurophysiol..

[10] Zuiderbaan W., Harvey B.M., Dumoulin S.O. (2012). Modeling center-surround configurations in population receptive fields using fMRI. J. Vis..

[11] Harvey B.M., Dumoulin S.O. (2011). The relationship between cortical magnification factor and population receptive field size in human visual cortex: constancies in cortical architecture. J. Neurosci..

